# Decreased Platelet Count in Patients Receiving Continuous Veno-Venous Hemofiltration: A Single-Center Retrospective Study

**DOI:** 10.1371/journal.pone.0097286

**Published:** 2014-05-13

**Authors:** Buyun Wu, Dehua Gong, Bin Xu, Qunpeng He, Zhihong Liu, Daxi Ji

**Affiliations:** National Clinical Research Center of Kidney Diseases, Jinling Hospital, Nanjing University School of Medicine, Nanjing, China; University of Kentucky, United States of America

## Abstract

**Background:**

A decreased platelet count may occur and portend a worse outcome in patients receiving continuous renal replacement therapy (CRRT). We aim to investigate the incidence of decreased platelet count and related risk factors in patients receiving CRRT.

**Methods:**

In this retrospective study, we screened all patients receiving continuous veno-venous hemofiltration (CVVH) at Jinling Hospital between November 2008 and October 2012. The patients were included who received uninterrupted CVVH for more than 72 h and had records of blood test for 4 consecutive days after ruling out pre-existing conditions that may affect the platelet count. Platelet counts before and during CVVH, illness severity, CVVH settings, and outcomes were analyzed.

**Results:**

The study included 125 patients. During the 3-day CVVH, 44.8% and 16% patients had a mild decline (20–49.9%) and severe decline (≥50%) in the platelet count,respectively; 37.6% and 16.0% patients had mild thrombocytopenia (platelet count 50.1–100×10^9^/L) and severe thrombocytopenia (platelet count ≤50×10^9^/L), respectively. Patients with a severe decline in the platelet count had a significantly lower survival rate than patients without a severe decline in the platelet count (35.0% versus 59.0%, P = 0.012), while patients with severe thrombocytopenia had a survival rate similar to those without severe thrombocytopenia (45.0% versus 57.1%, P = 0.308). Female gender, older age, and longer course of the disease were independent risk factors for a severe decline in the platelet count.

**Conclusions:**

A decline in the platelet count and thrombocytopenia are quite common in patients receiving CVVH. The severity of the decline in the platelet count rather than the absolute count during CVVH may be associated with hospital mortality. Knowing the risk factors for a severe decline in the platelet count may allow physicians to prevent such an outcome.

## Introduction

Thrombocytopenia is common (8.3–67.6%) in critically ill patients [1] and associated with increased mortality [2], [3]. The common risk factors for thrombocytopenia include sepsis, organ dysfunction, and severe illness. For critically ill patients with severe acute kidney injury, continuous renal replacement therapy (CRRT) is usually required [2], [3], but may increase the risk for thrombocytopenia and harm the patients [4]. However, there are far fewer studies regarding thrombocytopenia in CRRT than in hemodialysis (HD), although patients requiring CRRT are more critically ill than patients receiving HD.

The potential deleterious effect of HD on the platelet count can be attributed to consumption by circuit clotting, mechanical destruction, the bioincompatible reaction of the filter and heparin-induced thrombocytopenia (HIT). And a slight, transient decrease in the platelet count (typically 5–15%) was reported during the first 15–30 min of HD [5]. Because the duration of CRRT exceeds that of HD, it is reasonable to have concerns regarding the decline in the platelet count in patients receiving CRRT.

To determine the effect of hemofilters on the platelet count in CRRT, Mulder et al. [6] revealed a small, but significant decrease in the platelet count after a single pass through the filter. But sequential platelet counts had not been evaluated in patients undergoing CRRT. In contrast, CRRT may improve thrombocytopenia in septic patients by clearing inflammatory mediators that contribute to thrombocytopenia [7]. More importantly, severe decline in platelet counts is significantly associated with a worse outcome in acute kidney injury patients requiring renal replacement therapy [8], and CRRT may increase the risk for thrombocytopenia. Therefore we need to know the actual platelet count change in patients receiving long-term CRRT and explore the reasons for severe decline in the platelet count. To date, this issue has not been studied.

Thus, we designed this study to investigate incidence of the decline in the platelet count and thrombocytopenia in patients receiving CRRT. We also analyzed the effect of the decline in the platelet count on survival rate and associated risk factors.

## Methods

### Ethics Statement

The study was approved by the Ethics Committee of Jinling Hospital. The Ethics Committee waived the need for informed consent as the study was retrospective and the data were analyzed anonymously.

### Study Patients

All patients receiving continuous veno-venous hemofiltration (CVVH) at Jinling Hospital between November 2008 and October 2012 were screened. The patients were included who received uninterrupted CVVH for more than 72 h and had records of blood test for 4 consecutive days. The exclusion criteria included pre-existing conditions that may affect the platelet count except sepsis.

### CVVH Protocol

All patients received CVVH using Aquarius system (Baxter, Chicago, USA) or Multifiltrate system (Fresenius, Bad Homburg, Germany). Central venous catheterization was used for vascular access via the femoral or internal jugular vein. Blood flow was set at 150–230 mL/min. Bicarbonate-based replacement fluid was infused at a rate of 2 or 4 L/h through a pre-dilution route, which is the preferential CRRT mode at Jinling Hospital. Two types of filters, AV600 (polysulfone, 1.4 m^2^; Fresenius) and FX60 (polysulfone, 1.4 m^2^; Fresenius), were used at the discretion of the attending physician. The circuit was anticoagulated using regional citrate or systemic low molecular weight heparins (LMWHs). Citrate sodium was infused into patients with a high risk of bleeding at the rate of 21.6–28.8 mmol/h to achieve a circuit ionized calcium concentration <0.4 mmol/L [9]. And loading doses of 2000–5000 units of LMWHs were used in patients without an increased bleeding risk, with maintenance doses of 3–10 U/kg/h. The anti-Xa factor activity and anti-heparin/platelet factor 4 antibody were not monitored, as the tests are not routinely available in Jinling Hospital.

### Data Collection

Clinical and laboratory data were retrieved from the Jinling Hospital CRRT database and electronic medical records. The platelet counts and mean platelet volumes were recorded for 4 consecutive days (between 6 and 8 am); demographic, clinical, and biochemical data were also recorded. The Sequential Organ Failure Assessment (SOFA) score before CVVH and the Acute Physiology and Chronic Health Evaluation (APACHE) II score before and during CVVH were used for assessment of disease severity.

### Definitions

Thrombocytopenia and severe thrombocytopenia during CVVH were defined by platelet counts ≤100×10^9^/L and ≤50×10^9^/L, respectively. Mild and severe decline in platelet counts during CVVH were defined as a 20–49.9% reduction and a ≥50% reduction from the baseline platelet count, respectively. Acute kidney injury was diagnosed according to the KDIGO criteria [10]. Systemic inflammatory reaction syndrome and sepsis were defined based on 1992 consensus definitions [11]. The course of the disease was defined as the day of onset of the disease.

### Statistical Analysis

Based on the severity of the maximal decline in the platelet count from the baseline in 3-day CVVH, the patients were divided into three groups: ≥50% (severe decline); 20–49.9% (mild decline); and <20% (no decline). Similarly, based on the lowest platelet count in 3-day CVVH, the patients were divided into three groups: ≤50×10^9^/L (severe thrombocytopenia); 50.1–100×10^9^/L (mild thrombocytopenia); and >100×10^9^/L (normal platelet counts).

Continuous variables are presented as mean ± standard deviation (SD) for normal distribution, or as median (25^th^, 75^th^ percentile) for non-normal distribution. And the comparisons between groups were made using Student’s t-test for normal distribution or Wilcoxon rank sum test for non-normal distribution. Categorical variables are expressed as numbers and percentages, and comparisons between groups were made using Chi-square test.

Survival curves of the groups were generated using the Kaplan-Meier method, and comparisons were made using the log-rank test. Univariate and multivariate adjusted Cox regression analyses were performed to determine whether or not a severe decline in the platelet count was an independent risk factor for mortality. To explore the potential risk factors for a severe decline in the platelet count and severe thrombocytopenia, univariate logistic regression analyses were performed, and the variables that were found to be statistically significant (P<0.05) in the univariate analysis were included in multivariate analyses with the stepwise selection method. In these multivariate logistic analyses, adjustments were made for patient-related factors (age, gender), disease severity-related factors (APACHE II score, SOFA score, sepsis, hypotension, course of the disease, and thrombocytopenia before CVVH) and CRRT-related factors (filters, time of circuit clotting, anticoagulation, blood flow, and dose). Furthermore, receiver operating characteristic (ROC) analyses were performed to compare the predictive accuracy of risk factors for a severe decline in the platelet count and severe thrombocytopenia, and the areas under the curve (AUC) were calculated. The Hosmer–Lemeshow goodness-of-fit tests were used to evaluate the models’ calibration [12]. Data were analyzed using SAS 9.2 (SAS Institute, Cary, NC, USA). A two-sided P<0.05 was considered statistically significant.

## Results

### Patient Enrollment

**Figure 1:**
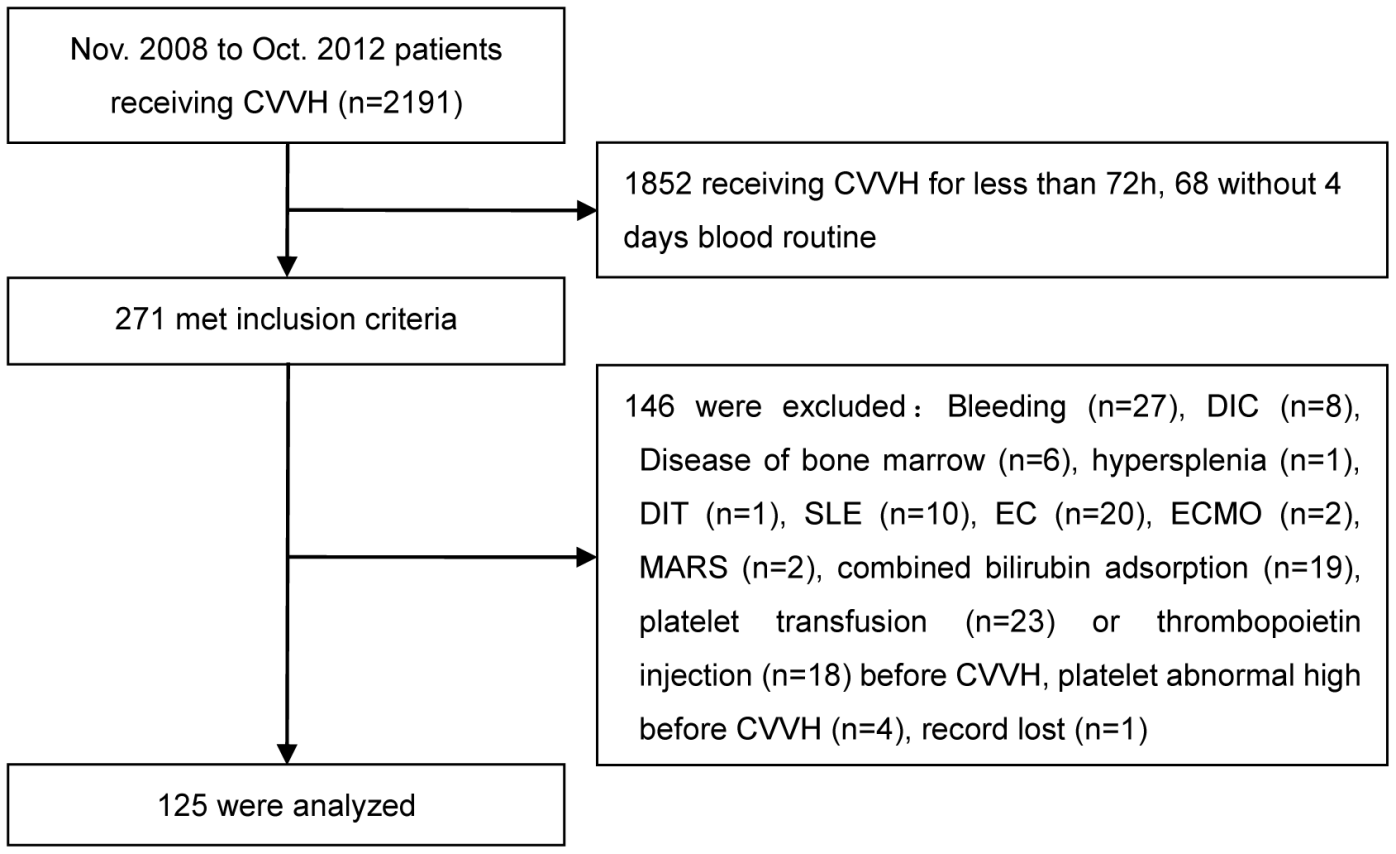
Flow chart of study participation. DIC: disseminated intravascular coagulation; DIT: drug-induced thrombocytopenia; SLE: systemic lupus erythematosus; EC: extracorporeal circulation; ECMO: extracorporeal membrane oxygenation; MARS: molecular adsorbent recirculating system.

A total of 2191 inpatients receiving CRRT were screened and 271 patients treated by CVVH more than 72 h with full data of platelet counts were enrolled. One hundred forty-six patients were excluded due to pre-existing conditions that may have affected the platelet counts, thus 125 patients were entered into the final analysis (Figure 1).

### Population Characteristics

The patients consisted of 97 males and 28 females, with a media age of 49 (39, 67) years. The primary diseases included severe acute pancreatitis (50.4% [63/125]), abdominal infections (25.6% [32/125]), trauma (6.4% [8/125]), pneumonia (8% [10/125]), and other diseases (9.6% [12/125]). The media SOFA score before CVVH was 7 (6, 10) and the mean highest APACHE II score before and during CVVH was 17.6±6.5. During the 72-h CVVH, the media time of hemofilter clotting was 2 (1, 2), and the media number of blood units transfused was 0 (0, 3) U. Nine patients had thrombopoietin injection and seven patients had platelet transfusion due to thrombocytopenia. The overall 90-day mortality was 43.2% (54/125).

### Changes in Platelet Counts and Mean Platelet Volume

The mean platelet count on the second and third day after CVVH initiation (Table 1, Figures S1–S2) was significantly decreased compared to that before treatment (P<0.05), while the mean platelet volume (MPV) remained unchanged. The platelet count on the third day was reduced by 19.58±54.76×10^9^/L (11±38.6%) compared to that before treatment (Figure S3–S4). During the 3-day CVVH, the incidence of platelet counts reduced by ≥20% and ≥50% was 60.8% and 16.0%, respectively. The incidence of thrombocytopenia and severe thrombocytopenia was 53.6% and 16.0%, respectively. The changes in the sequential platelet counts in different subgroups are shown in Table S1.

**Table 1:** The change in the platelet count and associated parameters and during continuous veno-venous hemofiltration (n = 125).

	Pre-CVVH	Day 1	Day 2	Day 3	During 3–day CVVH
Platelet count (10^9^/L)	138(99,175)	121(81,162)	107(71,155)*	114(71,151)*	–
Mean platelet volume (fl)	11.5±1.7	11.4±1.6	11.6±1.6	11.2(10.3,12.8)	–
Thrombocytopenia	33(26.4%)	47(37.6%)	53(42.4%)*	55(44.0%)*	67(53.6%)
Severe thrombocytopenia	5(4.0%)	7(5.6%)	8(6.4%)	15(12.0%)*	20(16.0%)
PCs reduction ≥50%	–	3(2.4%)	9(7.2%)	18(14.4%)	20(16.0%)
PCs reduction 20–49.9%	–	37(29.6%)	56(44.8%)	39(31.2%)	56(44.8%)
Change of PCs <20%	–	80(64.0%)	48(38.4%)	41(32.8%)	45(36.0%)
PCs increase ≥20%	–	5(4.0%)	12(9.6%)	27(21.6%)	4(3.2%)

*P<0.05 compared with pre-CVVH. CVVH: continuous veno-venous hemofiltration; PCs: platelet counts.

### Association between Decline in the Platelet Count and Thrombocytopenia on Survival Rate

**Figure 2:**
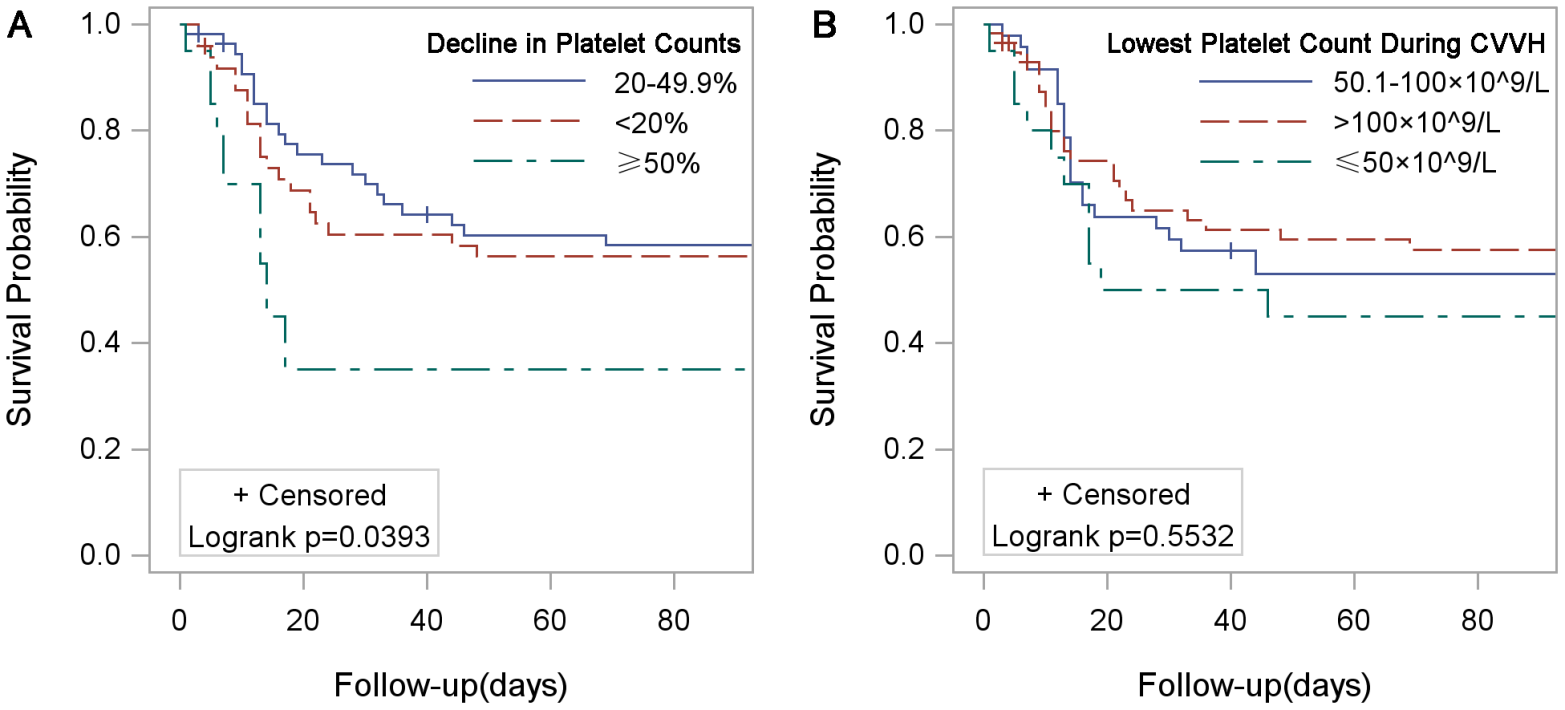
Kaplan-Meier plots for cumulative 90-day survival. Patients with a severe decline in the platelet count (≥50%) in 3-day CVVH had a worse 90-day survival than those with a mild decline in the platelet count (20–49.9%) or with no decline in the platelet count (<20%; Figure 2A). Patients with severe thrombocytopenia (Lowest Platelet count ≤50×10^9^/L) in 3-day CVVH had a similar 90-day survival compared to those with mild thrombocytopenia (Lowest Platelet count 50.1–100×10^9^/L) or with normal platelet counts (Lowest Platelet count>100×10^9^/L; Figure 2B). CVVH: continuous veno-venous hemofiltration.

As mentioned above, the patients were divided into three groups: ≥50% (severe decline); 20–49.9% (mild decline); and <20% (no decline). The baseline characteristics of each group are shown in Table 2. The differences in age, APACHE II scores, incidence of hypotension, cardiovascular and nervous SOFA scores, and course of the disease among the three groups were significant. The survival rates of the three groups were significantly different (Figure 2A; P = 0.039). More specifically, patients with a decline in the platelet count ≥50% had a significantly lower survival rate than other groups of patients (35.0% versus 59.0%, P = 0.012; Figure S5).

**Table 2:** Baseline characteristics of the study population.

	All patients(n = 125)	PCs reduction≥50% (n = 20)	PCs reduction20–49.9% (n = 56)	PCs reduction<20% (n = 49)	P-value
Female gender (%)	28(22.4)	6(30)	12(21.4)	10(20.4)	0.670
Age (y)	49(39,67)	67.4±17.1	50.6±17.6†	46.8±17.4†	<0.001
APACHE II score	17.8±6.5	21.4±4.7	17.8±6.5*	16(12,20) †	0.003
Sepsis	77(61.6)	15(75)	37(66.1)	25(51.0)	0.118
Hypotension	48(38.4)	13(65)	16(28.6) †	19(38.8)*	0.016
Mechanical Ventilation	80(64.0)	14(70)	37(66.1)	29(59.2)	0.637
Diabetes mellitus	20(16.0)	4(20)	7(12.5)	9(18.4)	0.623
Mean SOFA score	7(6,10)	8.8±3.6	7(4,9)	7(6,8)	0.171
Respiratory score	2(2,3)	2(1.5,3)	2(2,3)	2(2,3)	0.736
Liver score	1(0,2)	1(0,1.5)	0.5(0,2)	1(0,2)	0.572
Cardiovascular score	0(0,2)	1.5(0,3)	0(0,1)†	0(0,1)†	0.011
Nervous score	0(0,1)	2(1,3)	0(0,1)†	0(0,1)†	<0.001
CRRT indication					
AKI	93(74.4)	15(75)	44(78.6)	36(73.5)	0.383
SIRS	72(57.6)	9(45)	34(60.7)	31(63.3)	0.360
Electrolyte disturbance	16(12.8)	3(15)	8(14.3)	5(10.2)	0.489
Fluid overload	11(8.8)	1(5)	3(5.4)	7(14.3)	0.223
Azotemia	38(30.4)	6(30)	20(35.7)	12(24.5)	0.462
Hemoglobin (g/L)	96(83,116)	111.6±24.6	91.5(82.5,114)	95(82,110)	0.092
Platelet count (10^9^/L)	138(99,175)	134.4±43.8	139.5(99.5,177)	132(95,180)	0.898
Blood urea nitrogen (mmol/L)	17.3(9.8,25.5)	18.8±9.4	16.0(9.2,26.7)	17.3(10.0,24.2)	0.920
Creatinine (µmol/L)	206(99,412)	168(103,232)	210(88,419)	282(128,479)	0.132
Albumin (g/L)	32.6(29.9,35.6)	33.0±5.5	32.7(31.2,35.8)	31.9(29.9,34.9)	0.573
Total bilirubin (µmol/L)	22.0(13.0,43.1)	22.4(10.2,45.9)	18.2(13.0,42.2)	28.2(13.7,43.1)	0.742
Serum sodium (mmol/L)	142(137,147)	144(139,146)	143(137,149)	141(137,144)	0.174
Course of the disease (days)	7(3,19)	16(8,40)	5(3,19)	7(4,14)*	0.045
Blood flow (mL/min)	180(160–180)	180(160–180)	180(160–180)	180(160–180)	0.215

APACHE: acute physiology and chronic health Evaluation; AKI: Acute kidney injury; CRRT: continuous renal replacement therapy; MPV: mean platelet volume; PCs: platelet counts; SOFA: sequential organ failure assessment; SIRS: systemic inflammatory reaction syndrome.

*P<0.05 compared with PCs reduction ≥50%;

† P<0.01 compared with PCs reduction ≥50%.

Similarly, the patients were divided into three groups: ≤50×10^9^/L (severe thrombocytopenia); 50.1–100×10^9^/L (mild thrombocytopenia); and >100×10^9^/L (normal platelet counts). Interestingly, the survival rates among patients with severe thrombocytopenia, mild thrombocytopenia, and normal platelet counts were similar (P = 0.553; Figure 2B). And more specifically, patients with severe thrombocytopenia had a similar survival rate compared with patients without severe thrombocytopenia (45.0% versus 57.1%, P = 0.308; Figure S6).

Furthermore, the risk factors for mortality were calculated using Cox regression (Table 3). A severe decline in the platelet count was a risk factor for mortality based on univariate Cox regression (P = 0.015), but was not an independent risk factor based on multivariate adjusted Cox regression (P = 0.717).

**Table 3:** Univariate and multivariate adjusted Cox regression analyses of risk factors for mortality.

	Univariate		Multivariate	
	HR (95%CI)	P-value	Adjusted HR (95%CI)	P-value
Gender (reference: female)	0.708(0.357–1.404)	0.323	1.185(0.556–2.526)	0.668
Age (per 1-y increment)	1.018(1.004–1.033)	0.010	0.995(0.978–1.012)	0.995
Sepsis	4.255(2.079–8.709)	<0.001	4.456(2.209–7.787)	<0.001
Hypotension	2.130(1.259–3.603)	0.005	0.814(0.424–1.561)	0.535
APACHE II score (per 1 point increment)	1.122(1.073–1.174)	<0.001	1.102(1.040–1.167)	0.001
SOFA score (per 1 point increment)	1.238(1.135–1.351)	<0.001	1.155(1.038–1.287)	0.008
Platelet counts reduction ≥50%	2.162(1.160–4.031)	0.015	1.142(0.558–2.337)	0.717

Adjusted factor is gender. APACHE: acute physiology and chronic health Evaluation; SOFA: sequential organ failure assessment.

### Risk Factors for a Severe Decline in the Platelet Count and Severe Thrombocytopenia

Univariate logistic regression analysis indicated that older age, APACHE II score, SOFA score, hypotension, thrombocytopenia prior to CVVH, and a longer course of the disease are potential risk factors for a severe decline in the platelet count and thrombocytopenia (Table 4). Furthermore, multivariate regression analysis revealed that older age (OR = 1.05, 95% CI = 1.02–1.09, P = 0.002) and longer course of disease (OR = 1.02, 95% CI = 1.00–1.04, P = 0.028) were the independent risk factors for a severe decline in the platelet count (Table 5). After adjustment for potential confounders, only female gender (adjusted OR = 5.57, 95% CI = 1.14–27.34, P = 0.034), older age (adjusted OR = 1.05, 95% CI = 1.01–1.10, P = 0.021), and longer course of the disease (adjusted OR = 1.04, 95% CI = 1.01–1.06, P = 0.008) remained independent risk factors for a severe decline in the platelet count. A high APACHE II score (OR = 1.11, 95% CI = 1.01–1.22, P = 0.030) and thrombocytopenia prior to CVVH (OR = 7.41, 95% CI = 2.37–23.22, P<0.001) were independent risk factors for severe thrombocytopenia, and thrombocytopenia before CVVH (adjusted OR = 7.97, 95% CI = 1.98–32.02, P = 0.003) remained a risk factor after adjustment for potential confounders.

**Table 4:** Univariate analyses of risk factors for a severe decline in the platelet count and severe thrombocytopenia during 3-day continuous veno-venous hemofiltration.

Variable	Severe decline in platelet counts		Severe thrombocytopenia	
	OR(95% CI)	P-value	OR(95% CI)	P-value
Gender (female: male)	1.62(0.56–4.69)	0.377	0.84(0.26–2.76)	0.779
Age (y)	1.06(1.03–1.09)	<0.001	1.03(1.01–1.06)	0.011
Sepsis	2.08(0.70–6.15)	0.185	2.08(0.70–6.15)	0.185
Hypotension	3.71(1.36–10.14)	0.010	3.71(1.36–10.14)	0.010
APACHE II score	1.11(1.03–1.20)	0.008	1.13(1.04–1.22)	0.003
SOFA score	1.16(1.00–1.35)	0.054	1.23(1.06–1.43)	0.008
Times of hemofilter clotting during 3-day CVVH	1.28(0.85–1.92)	0.239	0.82(0.54–1.26)	0.361
Anticoagulation (LMWHs: citrate)	1.28(0.49–3.34)	0.610	0.79(0.30–2.10)	0.638
Blood flow	0.99(0.96–1.02)	0.430	0.99(0.96–1.02)	0.628
Filter (FX60:AV600)	0.83(0.30–2.24)	0.708	0.59(0.20–1.73)	0.339
Dose (4 L/h:2 L/h)	0.54(0.21–1.43)	0.218	0.69(0.27–1.81)	0.454
Volume change (balance: decreased)^a^	2.62(0.55–12.46)	0.226	2.38(0.50–11.41)	0.278
Volume change (increased: decreased)^a^	3.47(0.60–13.94)	0.163	4.41(0.79–24.50)	0.090
Thrombocytopenia pre-CVVH	0.66(0.20–2.12)	0.481	6.00(2.18–16.55)	<0.001
Receiving RRT before admission	0.46(0.13–1.70)	0.245	0.46(0.13–1.70)	0.245
Course of the disease (day)	1.02(1.01–1.04)	0.009	1.01(1.00–1.03)	0.111
Albumin	1.00(0.91–1.11)	0.913	1.04(0.95–1.14)	0.412

APACHE: acute physiology and chronic health Evaluation; OR = odds ratio; CI = confidence interval; CVVH: continuous veno-venous hemofiltration; RRT: renal replacement therapy; LMWHs: low molecular weight heparins; SOFA: sequential organ failure assessment.

a Decreased volume refers to average positive fluid balance less than 0 ml/d (insensible water loss was not included in the calculations); Increased volume refers to average positive fluid balance more than 1500 ml/d; volume balance refers to fluid balance in between.

**Table 5:** Multivariate analyses of independent risk factors for a severe decline in the platelet count and severe thrombocytopenia during 3-day continuous veno-venous hemofiltration.

Risk factors	Severe decline in platelet counts		Severe thrombocytopenia	
	Crude OR	Adjusted OR	Crude OR	Adjusted OR
	(95% CI)	(95% CI)	(95% CI)	(95% CI)
Age (y)	1.05(1.02–1.09)	1.05(1.01–1.10)	1.03(1.00–1.06)	1.02(0.98–1.06)
	P = 0.002	P = 0.021	P = 0.071	P = 0.417
Gender (female: male)	–	5.57(1.14–27.34)	–	1.12(0.23–5.55)
		P = 0.034		P = 0.886
APACHE II score	–	1.10(0.96–1.26)	1.11(1.01–1.22)	1.11(0.99–1.24)
		P = 0.159	P = 0.030	P = 0.063
Hypotension	2.54(0.84–7.73)	2.10(0.50–8.76)	–	2.19(0.52–9.21)
	P = 0.100	P = 0.310		P = 0.284
Course of the disease (day)	1.02(1.00–1.04)	1.04(1.01–1.06)	–	1.01(0.99–1.04)
	P = 0.028	P = 0.008		P = 0.379
Thrombocytopenia Pre-CVVH	–	0.25(0.04–1.43)	7.41(2.37–23.22)	7.97(1.98–32.02)
		P = 0.121	P<0.001	P = 0.003
AUROC	0.82(0.72–0.93)	0.89(0.80–0.98)	0.85(0.75–0.95)	0.86(0.76–0.95)
Goodness-of-fit test	χ^2^ = 8.595	χ^2^ = 12.183	χ^2^ = 9.326	χ^2^ = 2.928
	P = 0.378	P = 0.143	P = 0.316	P = 0.939

Adjusted factors include age, gender, APACHE II score, SOFA score, sepsis, hypotension, times of hemofilter clotting, anticoagulation, blood flow, dose, course of the disease and thrombocytopenia before CVVH. APACHE: acute physiology and chronic health Evaluation; AUROC: area under the receiver operating characteristic curve; CVVH: continuous veno-venous hemofiltration; OR = odds ratio.

Female gender, older age, and longer course of the disease were included in logistic regression multivariate analysis to calculate the AUC. The AUC of risk factors for a severe decline in the platelet count was 0.836(95% CI = 0.728–0.944, P<0.001; Figure S7). Similarly, the AUC of risk factors, including the APACHE II score and thrombocytopenia before CVVH, for severe thrombocytopenia was 0.833 (95% CI = 0.734–0.931, P<0.001; Figure S8).

## Discussion

In this retrospective study, 44.8% and 16% patients had a mild decline (20–49.9%) and severe decline (≥50%) in the platelet count during the 3-day CVVH, respectively; 37.6% and 16.0% patients had mild thrombocytopenia (platelet count 50.1–100×10^9^/L) and severe thrombocytopenia (platelet count ≤50×10^9^/L), respectively. The patients with a severe decline in the platelet count had a higher mortality rate compared to those without a severe decline in the platelet count. The risk factors for a severe decline in the platelet count included female gender, older age, and longer course of the disease.

Renal replacement therapy has been proposed as a risk factor for thrombocytopenia in critically ill patients in previous studies. In a retrospective study, Hamida et al. [13] reported that HD increased the risk for thrombocytopenia (HR = 2.30, 95% CI = 1.10–4.80) in liver transplantation patients. Crowther et al. [14] also demonstrated that dialysis (HR = 3.1; 95% CI = 1.2–7.8) during the ICU stay is an independent risk factor for thrombocytopenia. Platelet loss caused by filters has been clearly demonstrated by Mulder et al. [6]; specifically, the platelet count decreased by a mean of 2.32×10^9^/L after a single pass of blood through a filter. Indeed, this effect may cause a transient slight decrease in the platelet count (typically 5–15%) during the first 15–30 min of HD [5]. However, the sequential platelet counts were not evaluated in patients receiving CRRT. So we do not know subsequent changes of platelet counts in patients, and cannot distinguish which patient would suffer a severe decline in the platelet count in long-term CRRT. Our results showed a significant decline in the platelet count during CRRT and an increase in the incidence of severe thrombocytopenia could occur during a 3-day CVVH, especially in the elderly subgroup (≥60 years) or in patients with a higher APACHE II score (≥19 points; Table S1). In agreement with Valente’s study [8], in which 13.5% of patients had a platelet count reduction ≥60% during renal replacement therapy within the first 3 days, our study showed that the incidence of a severe decline in the platelet count during 3-day CVVH was as high as 16%.

However, it is unknown whether or not the severe decline in the platelet count is due to CRRT-, patient- or disease severity-related factors. CRRT-related factors, such as circuit clotting and blood flow [6], can be theoretically associated with a decline in the platelet count in patients. However, risk factor analyses showed that CRRT-related factors, including different filters (all were polysulfone filters), time of circuit clotting, anticoagulant (LMWHs use or not), blood flow, and CRRT dose, are not independent risk factors for a severe decline in the platelet count or severe thrombocytopenia in the present study. In contrast, the development of a severe decline in the platelet count during CRRT may be attributed to patient-related factors, such as female gender and older age, or disease severity-related factors like a longer course of the disease. Considering the fact that the change in the circulating platelet counts is based on platelet destruction, sequestration, and bone marrow production, we hypothesized that a continuous slight loss of platelets caused by CRRT may result in a severe decline in the platelet count in critically ill patients with bone marrow platelet production dysfunction [15]. Previous studies have shown that androgens can improve thrombocytopenia in hematology patients [16] and inhibit oxidative-stress-induced platelet aggregation [17], which suggests that female gender has a higher risk for a severe decline in the platelet count compared to male gender. Therefore, factors that may affect platelet production, such as older age, longer course of disease, and female gender, are more likely to be risk factors for a severe decline in the platelet count during CRRT. Further confirmatory studies are required to validate this hypothesis.

Thrombocytopenia can increase the risk of death in critically ill patients [1], [14], [18], [19], but it needs to be confirmed in those patients receiving CRRT who have a high incidence of decline in the platelet count and thrombocytopenia. The incidence of thrombocytopenia increases in patients undergoing CRRT, therefore it is probable that CRRT can harm patients through consumption of platelets. Our study demonstrated that the patients with a severe decline in the platelet count (≥50%) during a 3-day CVVH treatment had a higher risk of mortality compared to those with a mild or no decline in the platelet count. This confirms Valente’s findings that severe platelet count reduction (≥60%) is significantly associated with a worse outcome in acute kidney injury patients requiring renal replacement therapy (80.2% CRRT) [8]. However, our study did not show that a severe decline in the platelet count was an independent risk factor for mortality based on the multivariate adjusted Cox regression model. A probable explanation for this finding is that a severe decline in the platelet count indicates bone marrow dysfunction or an increase in platelet destruction by other causes than CRRT, both of which are predictive of more severe illness that can explain severe decline in the platelet count. In contrast, a mild decline in the platelet count and thrombocytopenia in the CVVH can be reversed when CVVH is discontinued. In brief, the patients with a severe decline in the platelet count during CRRT have worse outcomes and should be evaluated for other causes.

We can take some precautions to reduce platelet loss in critically ill patients receiving CRRT, especially in those at a high risk for a severe decline in the platelet count. First, because biocompatibility of the membrane is associated with decreased platelet count, cellulose membranes [18] and non-electron beam-sterilized hemofilters [20] were preferred. Second, maintaining the patency of the circuit [21] can reduce loss of blood including platelet. Third, we can use regional citrate anticoagulation in patients who are at risk for bleeding [22] and combined therapy consisting of unfractionated heparin plus tirofiban [23], [24]. Lastly, we can use LMWHs [25] or prostacyclin [26], [27] instead of unfractionated heparin to reduce the incidence of HIT, or use argatroban instead of heparins if HIT exists [20].

This study described the high incidence of platelet count reduction and thrombocytopenia, and associated risk factors during CVVH, thus serves as an incentive for prevention. However, several limitations should be recognized. First, this study was retrospective with a limited sample size; it could not demonstrate the effect of CVVH on the platelet count and activation markers. Second, we could not rule out the effect of HIT on decreasing the platelet count, although 110 (88.0%) patients in our study were first treated by CVVH for 72 h. Finally, we could not rule out the disturbance in platelet counts due to other causes, such as drawing blood in the intensive care unit. Further studies are needed to determine the relationship between the platelet count and activation marker changes during CRRT.

## Conclusions

In conclusion, a decline in the platelet count and thrombocytopenia are common in patients receiving CVVH. The severity of the decline in the platelet count rather than the absolute platelet count during CVVH may be associated with hospital mortality. The risk factors for a severe decline in the platelet count included female gender, older age, and longer course of the disease. We must identify these risks for a severe decline in the platelet count and take precautions to avoid such outcomes in the clinic.

## Supporting Information

Figure S1 Change of platelet count compared to baseline during 3-day continuous veno-venous hemofiltration.
(TIF)

Figure S2 Extent of change of platelet count compared to baseline during 3-day continuous veno-venous hemofiltration.
(TIF)

Figure S3 Distribution of change of the third day’s platelet count compared to baseline in 3-day continuous veno-venous hemofiltration.
(TIF)

Figure S4 Distribution of change rate of the third day’s platelet count compared to baseline in 3-day continuous veno-venous hemofiltration.
(TIF)

Figure S5 Comparison of survival rates between groups with and without decline in platelet count ≥50%.
(TIF)

Figure S6 Comparison of survival rates between groups with and without severe thrombocytopenia.
(TIF)

Figure S7 ROC curve for the model of risk factors predicting severe decline in the platelet count.
(TIF)

Figure S8 ROC curve for the model of risk factors predicting severe thrombocytopenia.
(TIF)

Table S1 Decline of the platelet count in third day compared to that of pre-CVVH: subgroup analysis.
(DOCX)
