# Supplementary figures and images for: Decreased Platelet Count in Patients Receiving Continuous Veno-Venous Hemofiltration: A Single-Center Retrospective Study
Source: PLoS One. 2014 May 13;9(5):e97286. doi: 10.1371/journal.pone.0097286 (PMC4019530; doi:10.1371/journal.pone.0097286)

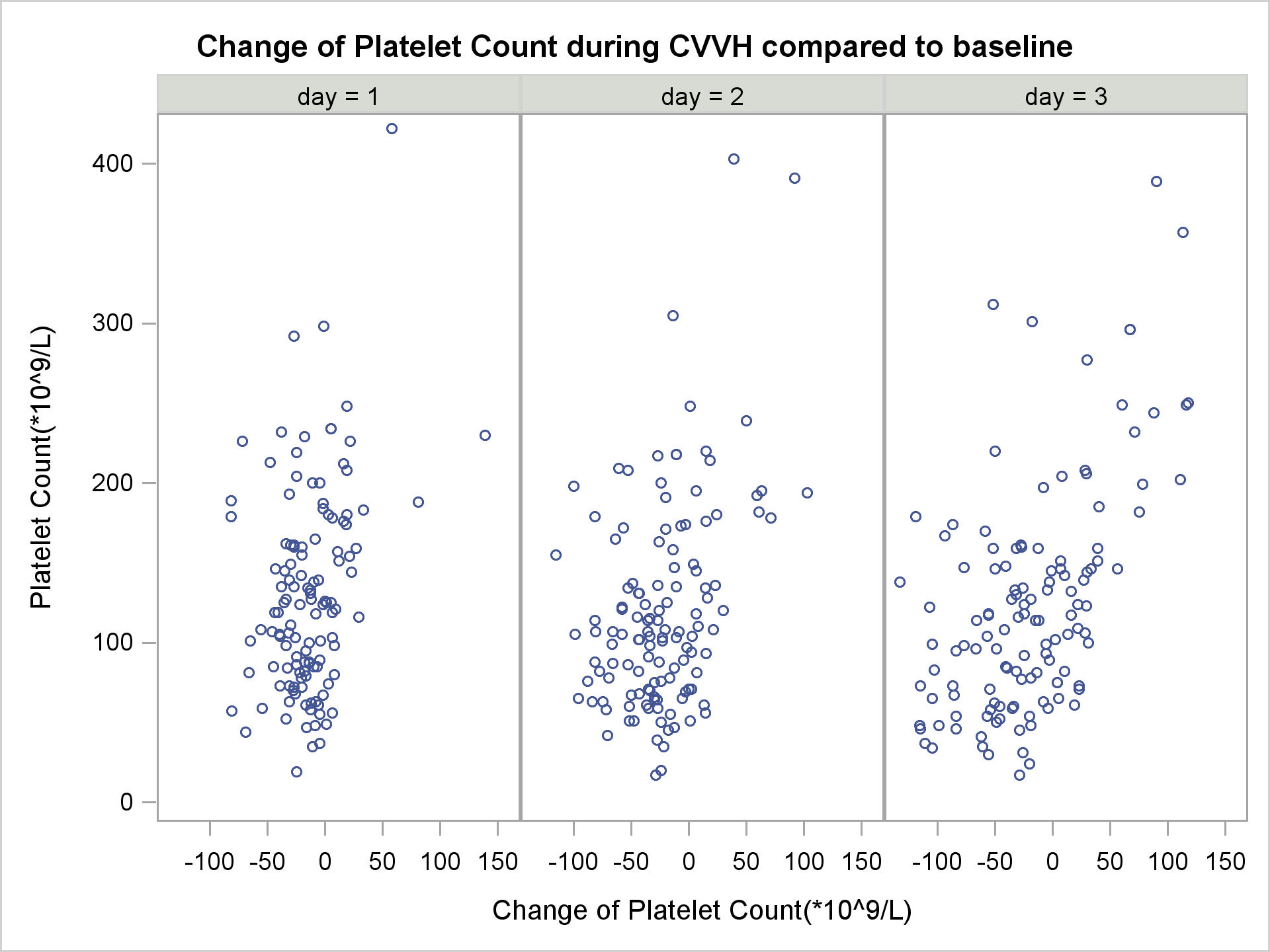

Supplement: Figure S1 — Change of platelet count compared to baseline during 3-day continuous veno-venous hemofiltration. (TIF) [file pone.0097286.s001.tif]

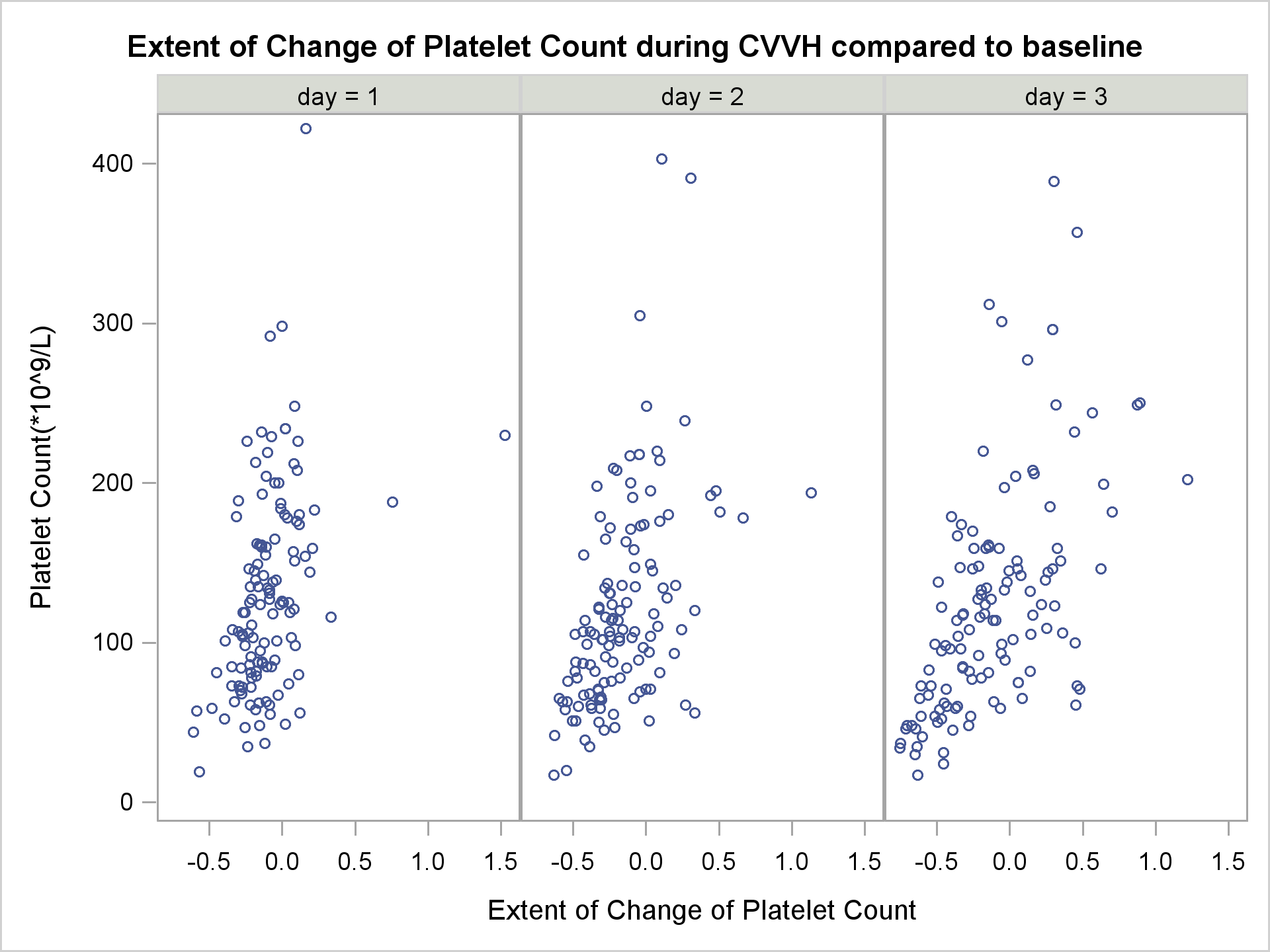

Supplement: Figure S2 — Extent of change of platelet count compared to baseline during 3-day continuous veno-venous hemofiltration. (TIF) [file pone.0097286.s002.tif]

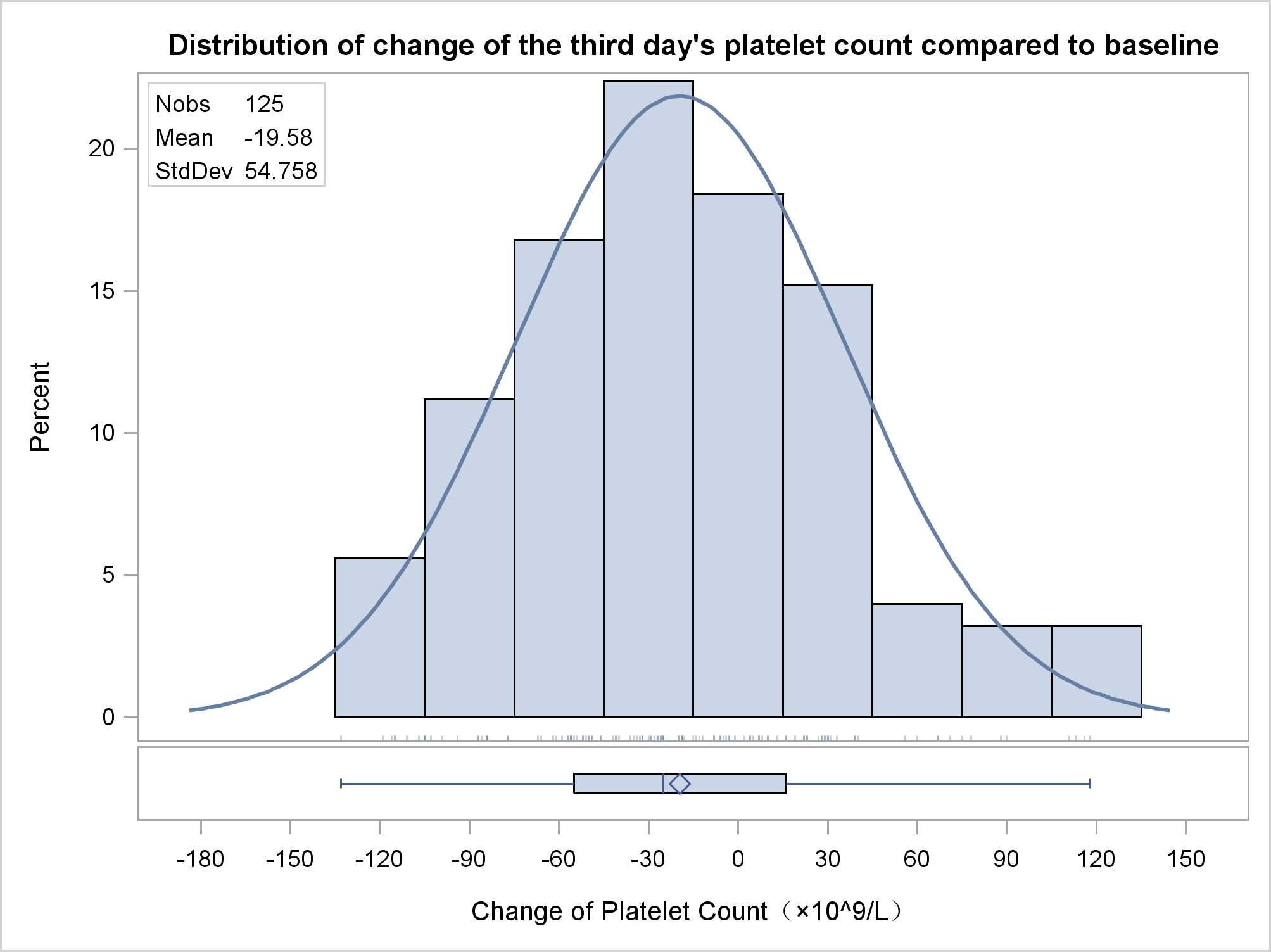

Supplement: Figure S3 — Distribution of change of the third day’s platelet count compared to baseline in 3-day continuous veno-venous hemofiltration. (TIF) [file pone.0097286.s003.tif]

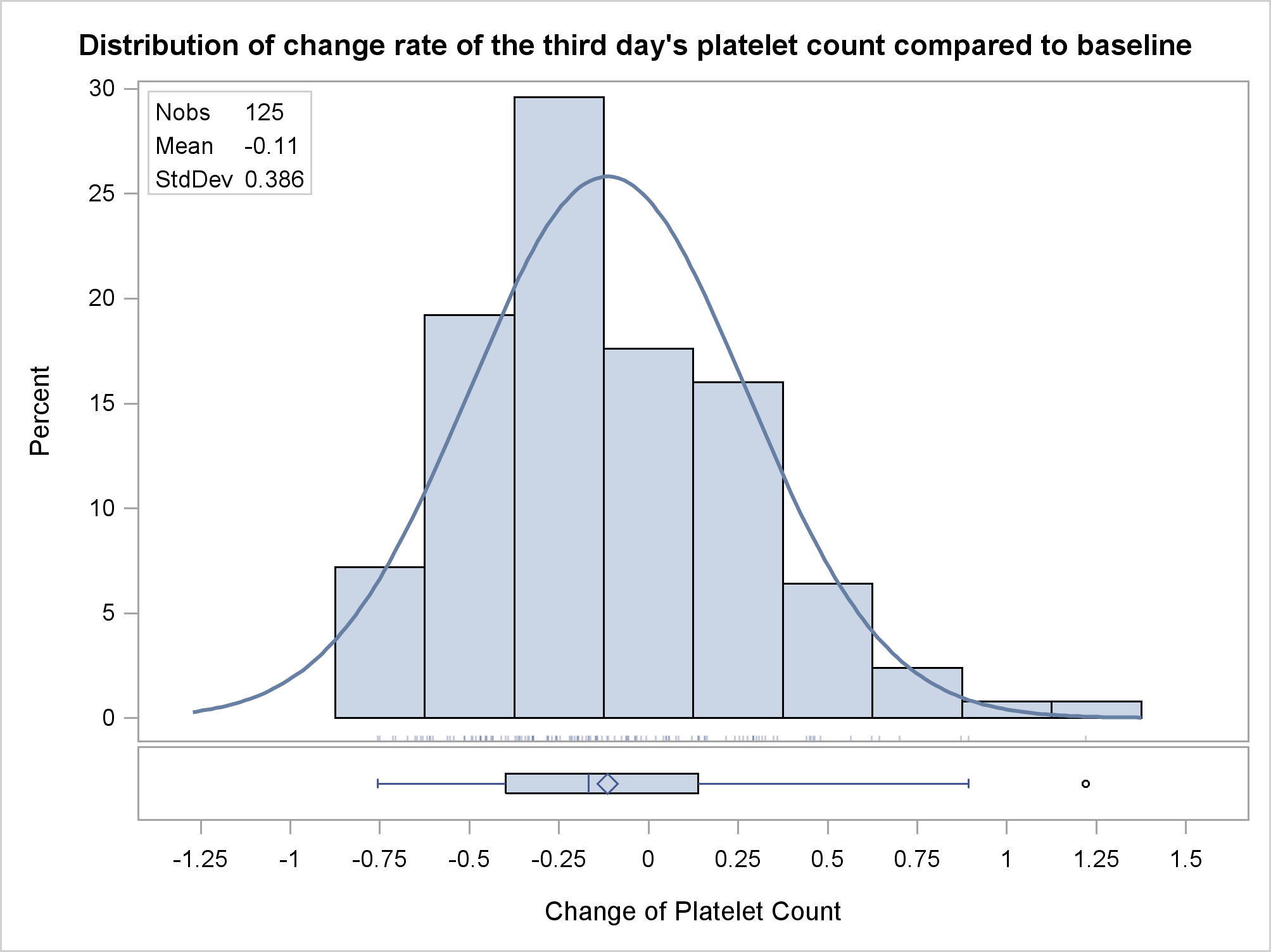

Supplement: Figure S4 — Distribution of change rate of the third day’s platelet count compared to baseline in 3-day continuous veno-venous hemofiltration. (TIF) [file pone.0097286.s004.tif]

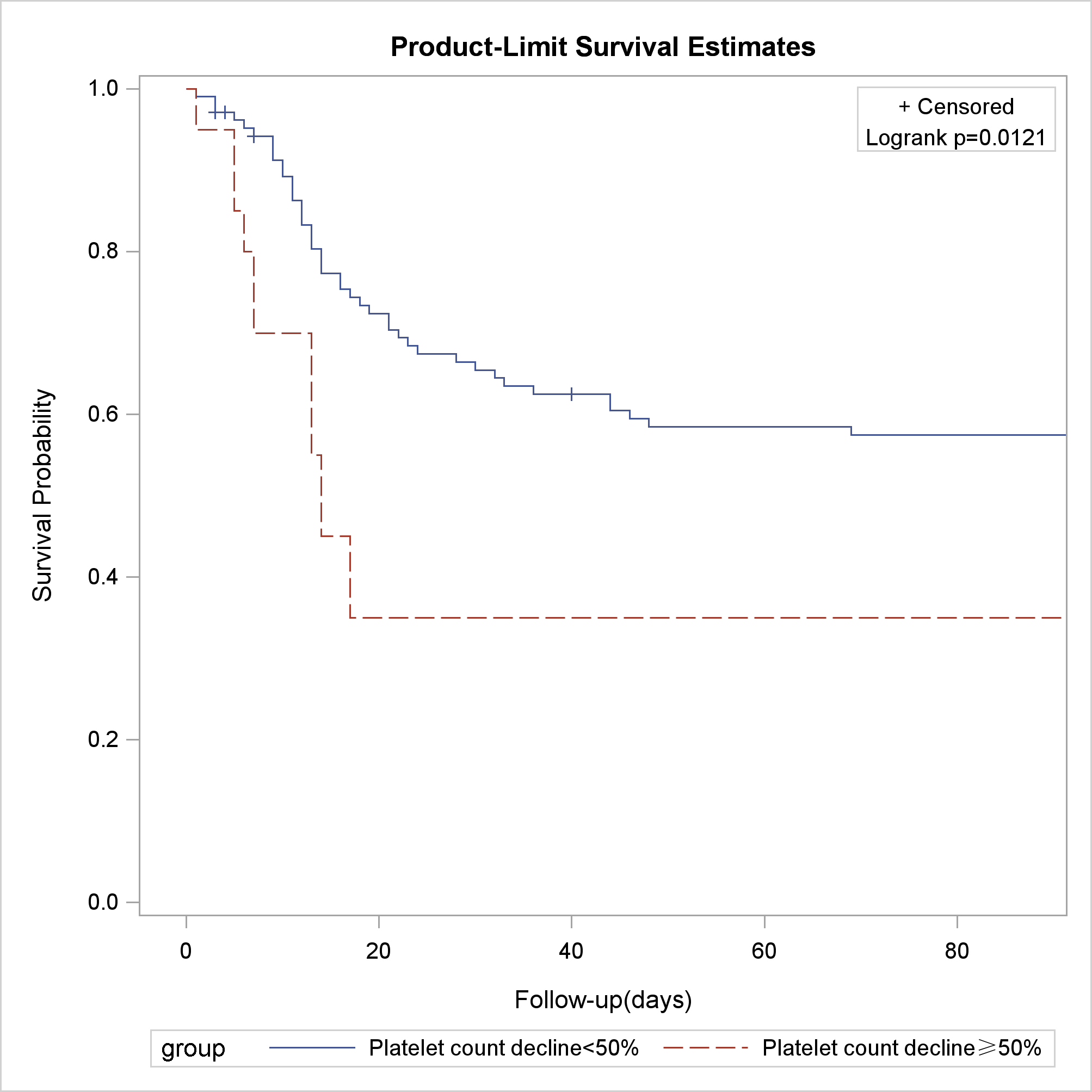

Supplement: Figure S5 — Comparison of survival rates between groups with and without decline in platelet count ≥50%. (TIF) [file pone.0097286.s005.tif]

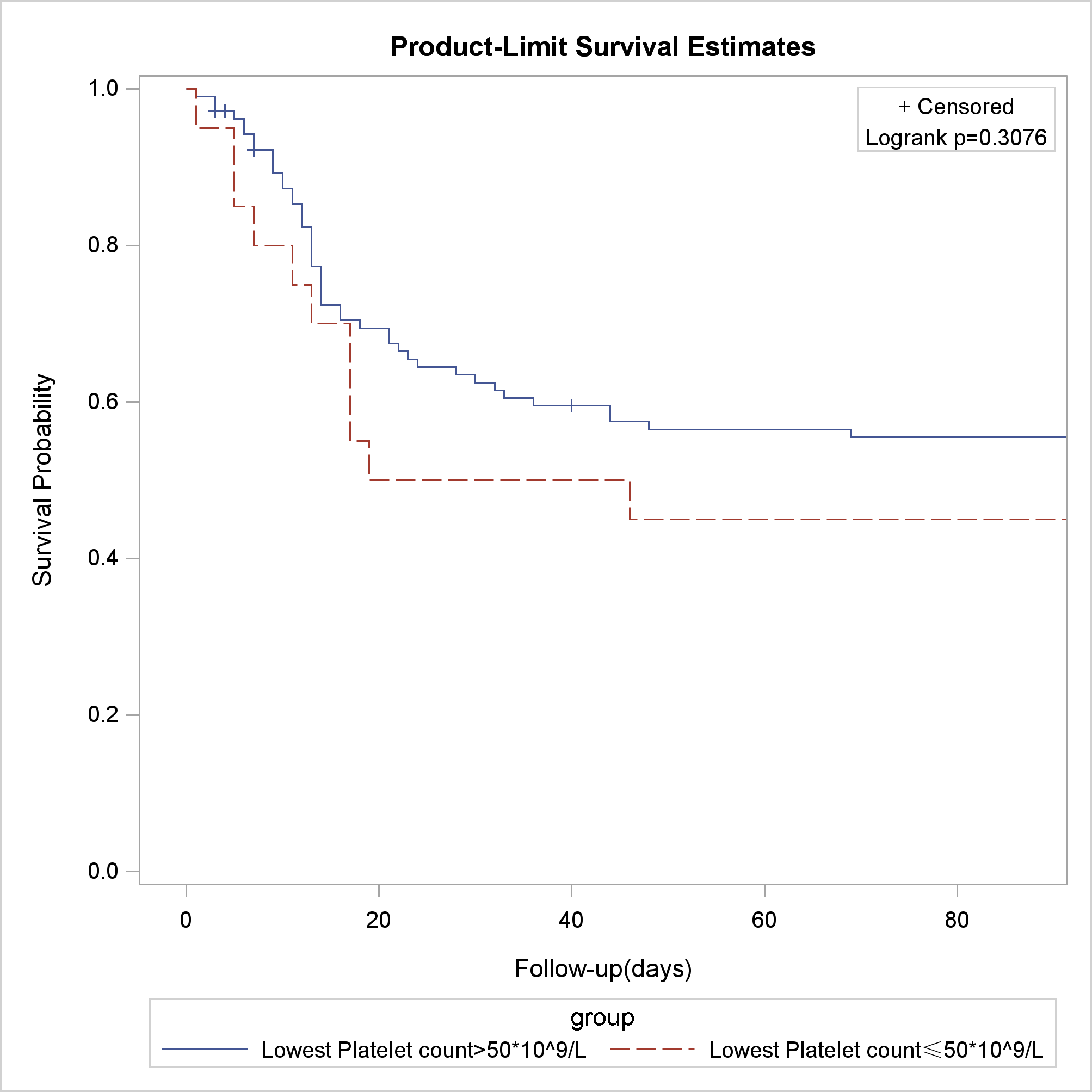

Supplement: Figure S6 — Comparison of survival rates between groups with and without severe thrombocytopenia. (TIF) [file pone.0097286.s006.tif]

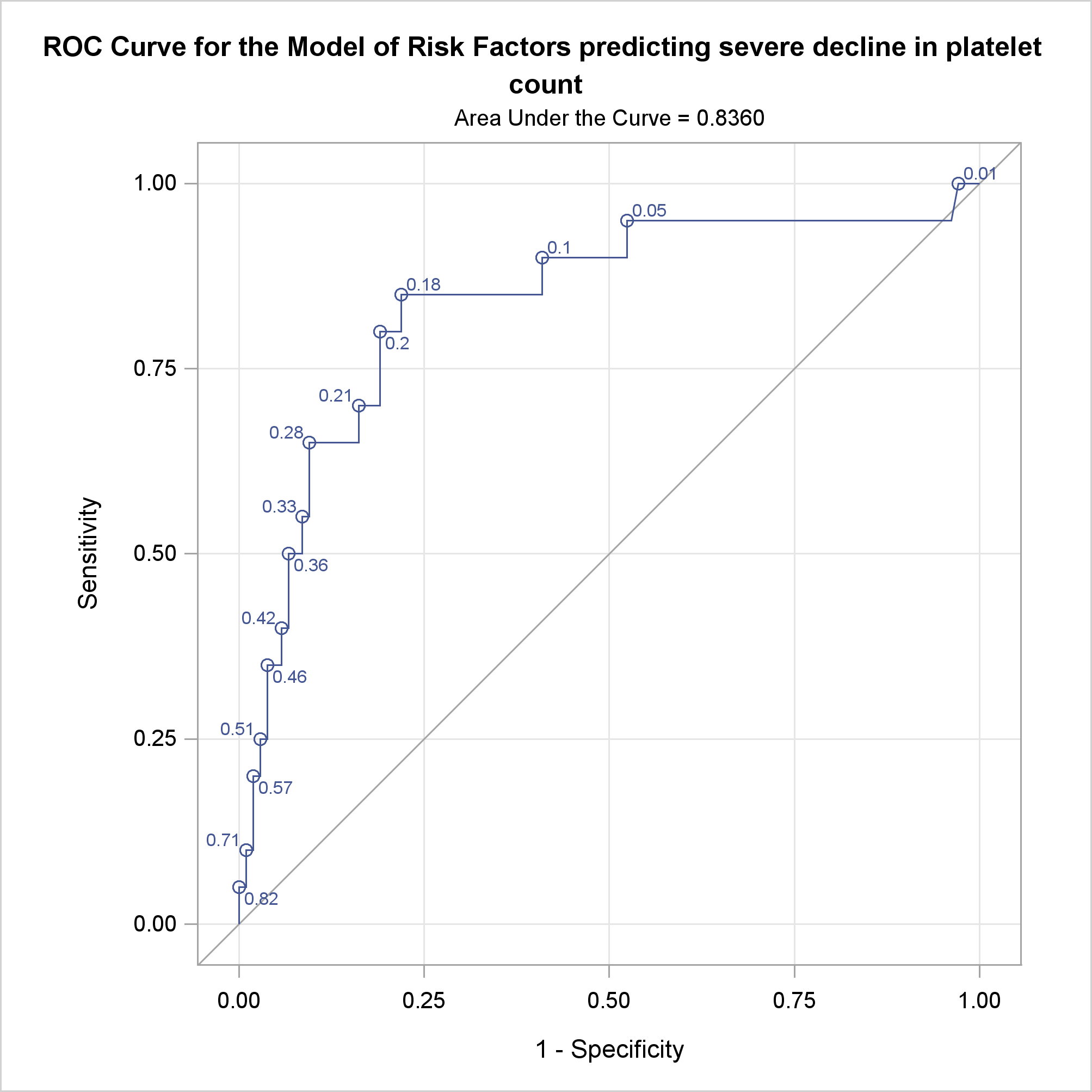

Supplement: Figure S7 — ROC curve for the model of risk factors predicting severe decline in the platelet count. (TIF) [file pone.0097286.s007.tif]

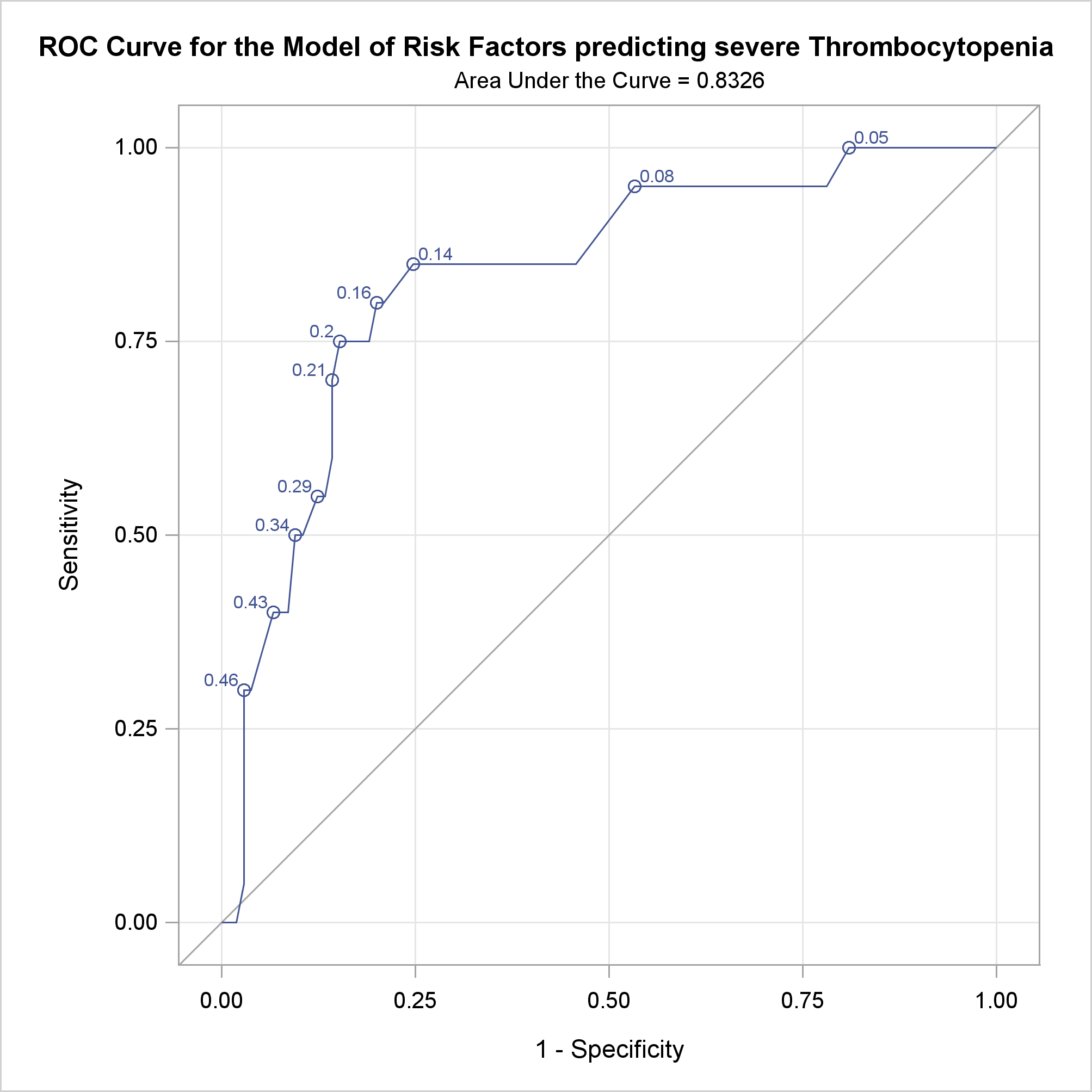

Supplement: Figure S8 — ROC curve for the model of risk factors predicting severe thrombocytopenia. (TIF) [file pone.0097286.s008.tif]
