# Supplementary material for: Decreased Platelet Count in Patients Receiving Continuous Veno-Venous Hemofiltration: A Single-Center Retrospective Study
Source: PLoS One. 2014 May 13;9(5):e97286. doi: 10.1371/journal.pone.0097286 (PMC4019530; doi:10.1371/journal.pone.0097286)
Supplement: Table S1 — Decline of the platelet count in third day compared to that of pre-CVVH: subgroup analysis. (DOCX) [file pone.0097286.s009.docx]

**Table S1** Decline of the platelet count in third day compared to that of pre-CVVH: subgroup analysis.

| Subgroup | Pre-CVVH PCs (10^9^/L) | P-value | Reduction in PCs (%) | P-value | Reduction rate in PCs (%) | P-value |
| --- | --- | --- | --- | --- | --- | --- |
| Age>=60（n=40） | 147.9±56.0 | 0.586 | 43.8±49.4 | **<0.001** | 30.3±31.8 | **<0.001** |
| Age<60（n=85） | 141.5±63.9 |  | 8.2±53.7 |  | 2.6±38.5 |  |
| APACHE II score>=19（n=58） | 149.4±64.8 | 0.321 | 36.6±53.1 | **0.001** | 25.2±34.4 | **<0.001** |
| APACHE II score<19（n=67） | 138.4±58.2 |  | 4.8±52.2 |  | -0.5±38.3 |  |
| Sepsis（n=77） | 152.5±66.3 | 0.026 | 26.5±58.0 | 0.072 | 16.0±69.6 | 0.097 |
| Non-sepsis（n=48） | 129.1±49.6 |  | 8.4±47.6 |  | 4.2±36.1 |  |
| hypotension（n=48） | 145.6±61.7 | 0.762 | 31.6±56.5 | 0.052 | 22.3±36.2 | **0.013** |
| Non-hypotension（n=77） | 142.2±61.5 |  | 12.1±52.6 |  | 4.7±39.7 |  |
| AKI（n=93） | 140.3±56.5 | 0.316 | 18.8±55.7 | 0.794 | 11.3±39.8 | 0.958 |
| Non-AKI（n=32） | 152.9±73.8 |  | 21.8±52.7 |  | 11.8±35.6 |  |
| thrombocytopenia（n=33） | 77.6±18.5 | <0.001 | 3.0±34.4 | 0.010 | 3.0±42.6 | 0.144 |
| Non-thrombocytopenia（n=92） | 167.2±53.5 |  | 25.5±59.4 |  | 14.5±36.9 |  |

AKI: acute kidney injury; APACHE: acute physiology and chronic health Evaluation; CVVH: continuous veno-venous hemofiltration; PCs: platelet counts.
